# Lost ampullary opening post endoscopic submucosal dissection: EUS-guided rendezvous shows the path

**DOI:** 10.1016/j.vgie.2024.09.004

**Published:** 2024-09-12

**Authors:** Darshan Parekh, Yohei Minato, Marina Kim, Shunya Takayanagi, Nao Takeuchi, Yuki Kano, Yuji Fujita, Ken Ohata

**Affiliations:** 1 Department of Endoscopy, Mumbai Institute of Gastroenterology, Mumbai, Maharashtra, India; 2 Department of Endoscopy, NTT Medical Center Tokyo, Tokyo, Japan; 3 Division of Gastroenterology & Hepatology, Saint Louis University School of Medicine, Saint Louis, Missouri, USA; 4 Department of Hepato-Biliary-Pancreatic Medicine, NTT Medical Center Tokyo, Tokyo, Japan

## Introduction

Endoscopic submucosal dissection (ESD) in the duodenum now has acceptable outcomes of 94.8% en bloc resection rate and 6.8% adverse events by highly experienced endoscopists.^1^ Ampullary ESD is furthermore technically challenging, requiring experienced hands. After ampullary resection, drainage of the common bile duct (CBD) and pancreatic duct (PD) is essential to prevent pancreatitis^2^ and delayed bleeding and perforation.^3^ When the ducts are inaccessible the traditional way, EUS-guided rendezvous (EUS-RV) can salvage the situation.^4^ We present a case where post-ampullary ESD and identification of CBD and PD openings were not possible, and drainage was achieved by a EUS-RV technique (Video 1, available online at www.videogie.org).

## Case

**Figure 1:**
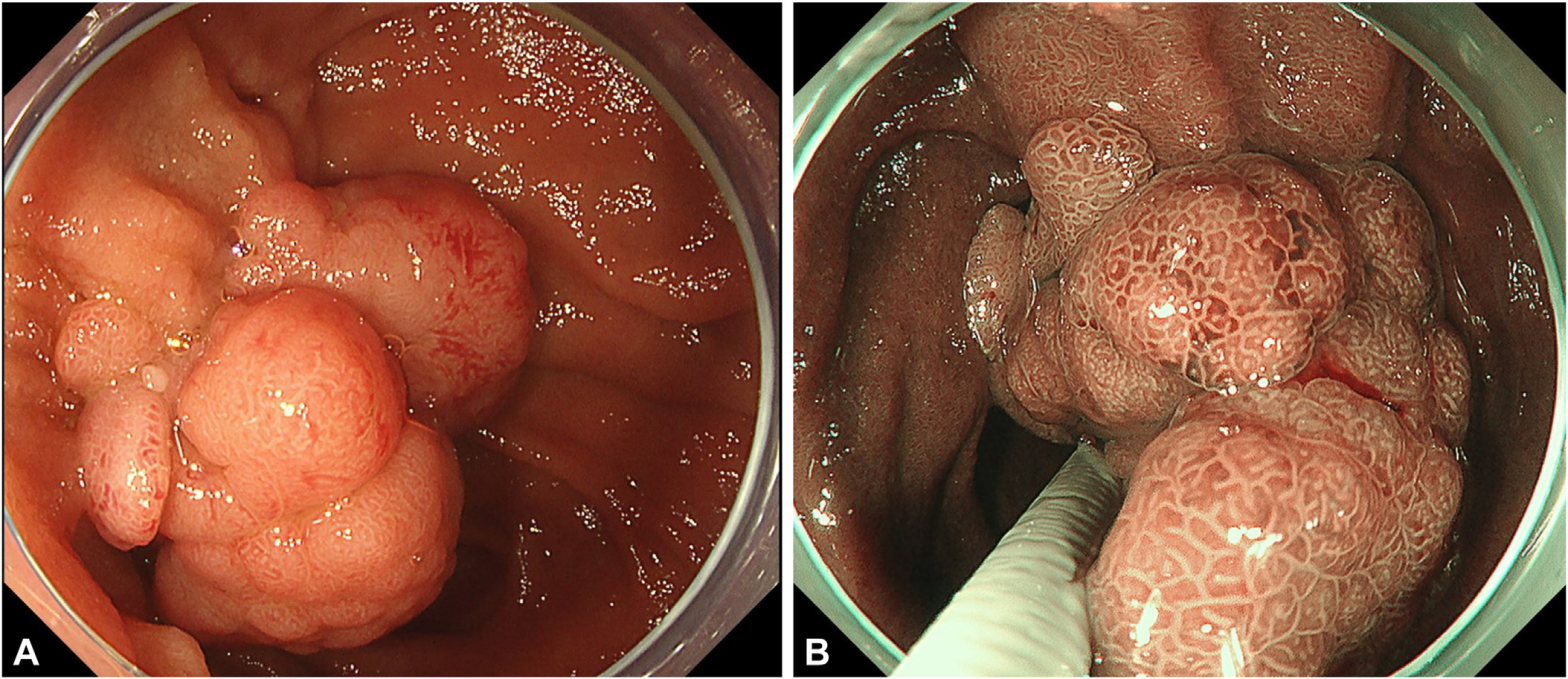
Ampullary tumor on forward-viewing endoscope. **A,** White light. **B,** Narrow-band imaging.

**Figure 2:**
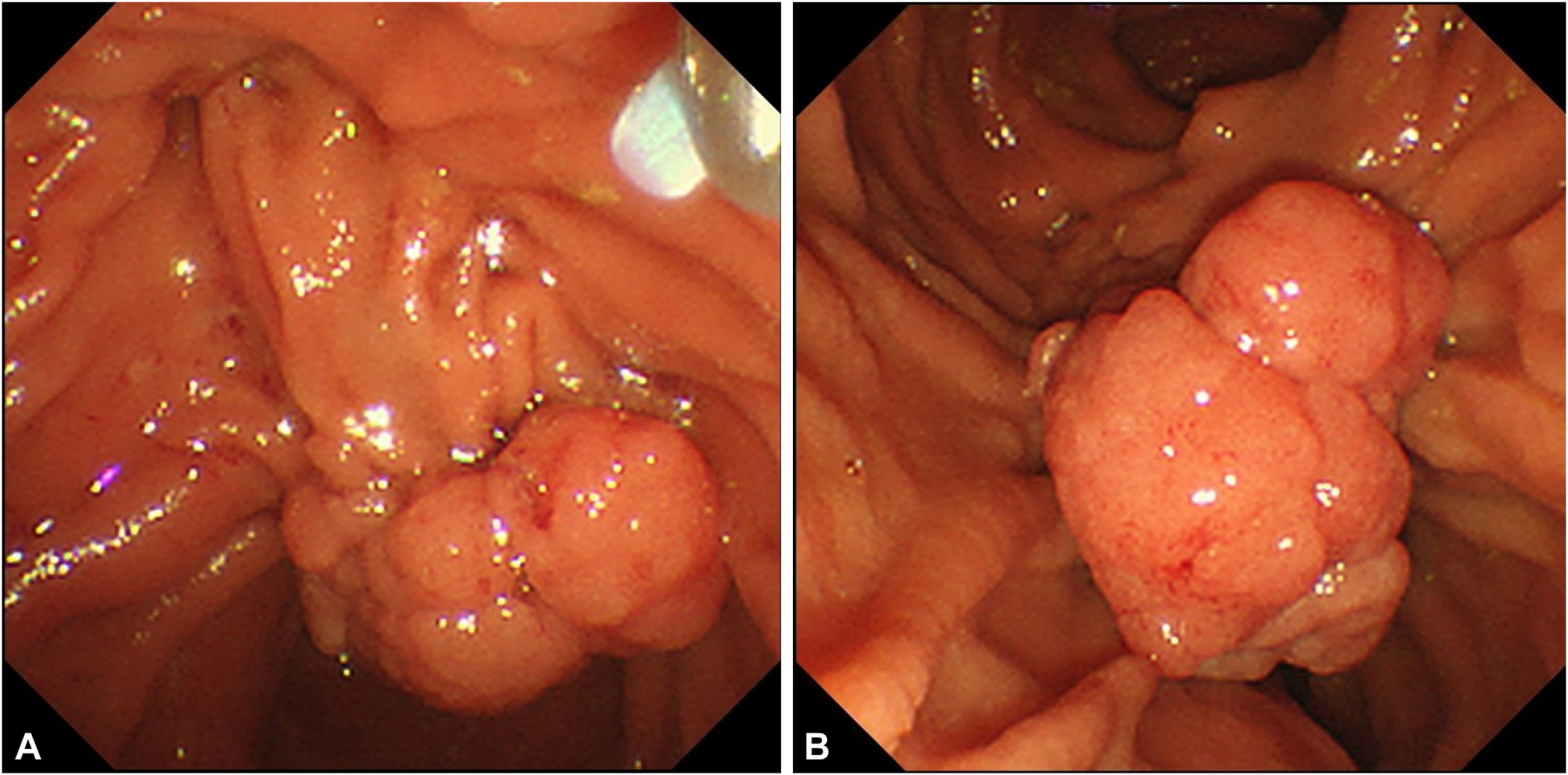
Ampullary tumor on side-viewing endoscope.

**Figure 3:**
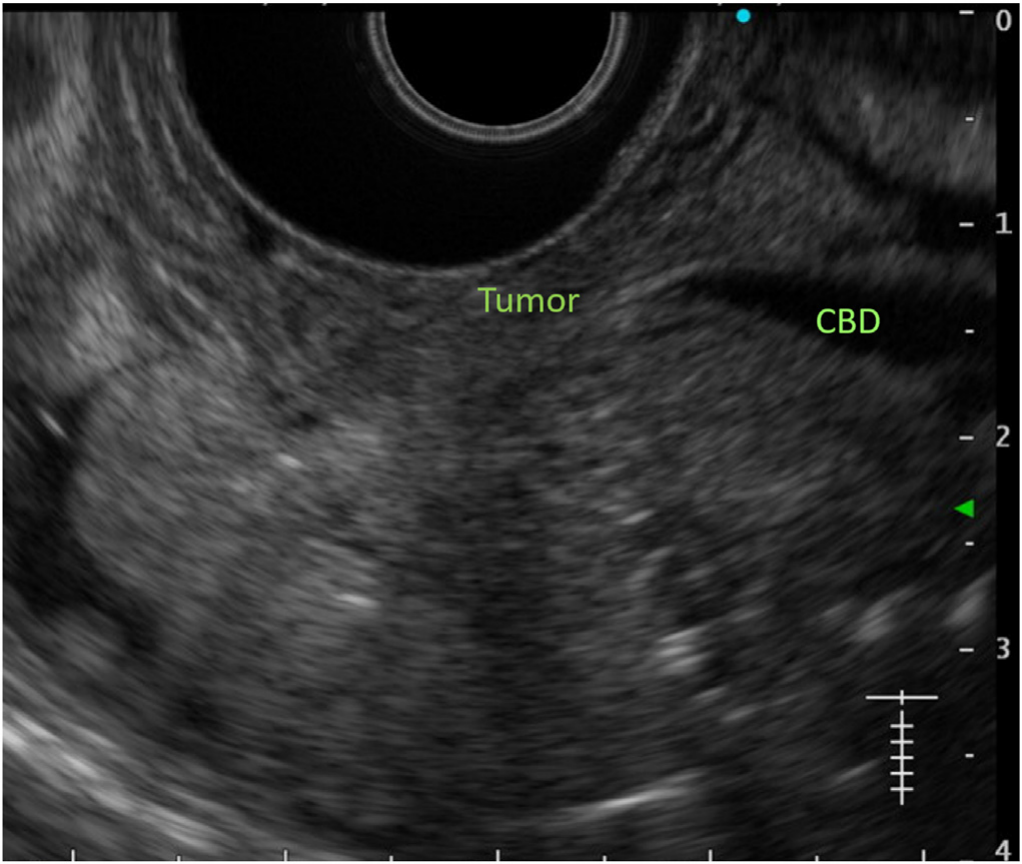
EUS showing no involvement of common bile duct (CBD) or pancreatic duct by the tumor.

An 80-year-old man was referred to our center for management of a duodenal tumor detected incidentally on upper endoscopy. The descending duodenal tumor was a protruding mucosal lesion of around 30 mm in size with a thick stalk, and lobular head with regular surface pattern. The ampulla could not be identified separately by forward- (Fig. 1) or side-viewing endoscope (SVE) (Fig. 2). EUS showed no involvement of CBD or PD (Fig. 3). Endoscopic resection under general anesthesia was planned for the patient. En bloc resection was planned as there is a high reported recurrence rate for piecemeal resection in duodenal lesions of more than 30 mm.

## Procedure

**Figure 4:**
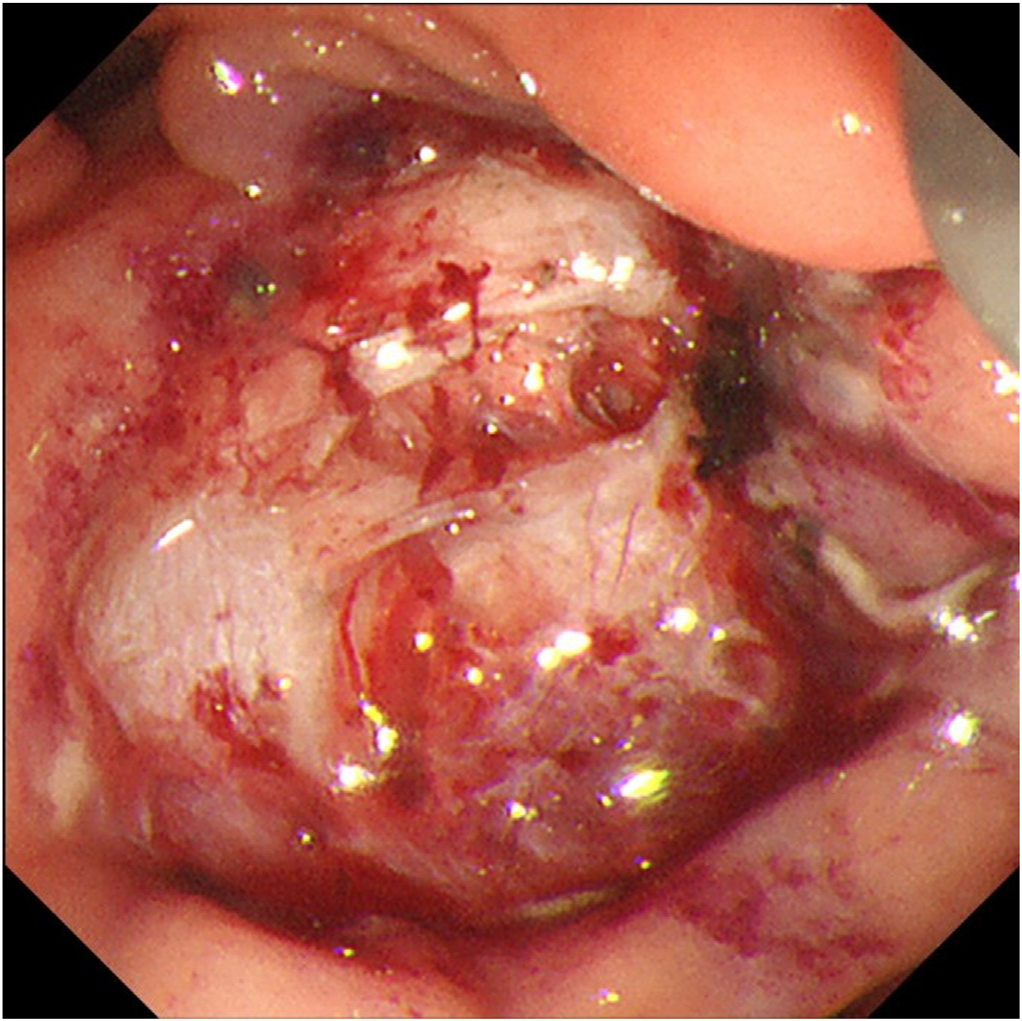
Post–endoscopic submucosal dissection ulcer with muscle defect. No other opening visible.

**Figure 5:**
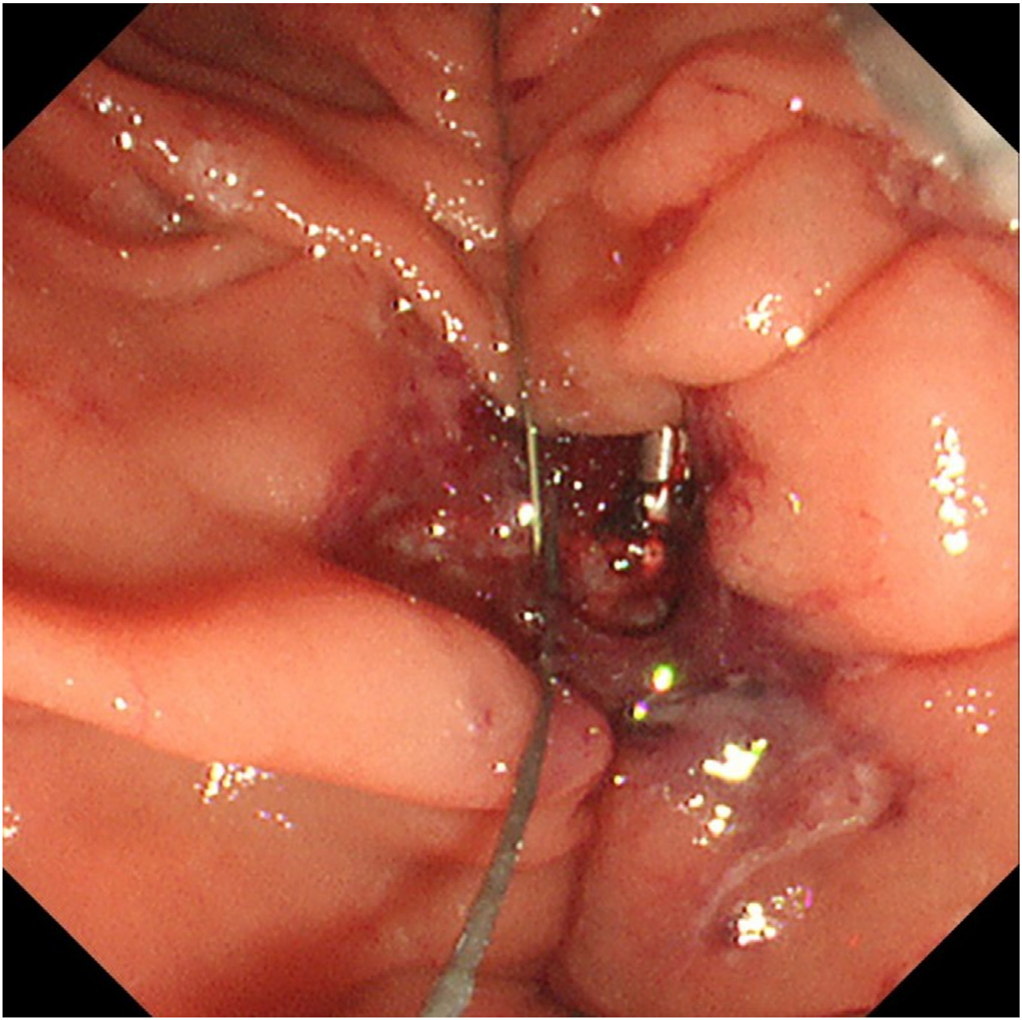
Guidewire from the common bile duct by EUS-guided rendezvous seen at the edge of the ulcer.

Resection by SVE was not possible because of the location. Using the forward-viewing endoscope, we initially tried to ligate the stalk. But complete ensnarement of the tumor could not be confirmed as the distal side could not be visualized. Hence, we decided to resect the tumor by ESD. Clip and line traction with electrosurgical generator (VIO3; Erbe, Tübingen, Germany) settings Endocut I, Effect 2, Interval 2, and Duration 2 were used to overcome the challenges of location, size, and submucosal fibrosis. Post ESD, the visualized opening in the ulcer (Fig. 4) assumed to be the ampullary opening turned out to be a muscle defect. No other opening could be identified. The muscle defect was closed with a hemostatic clip, and it was decided to conduct an EUS-RV. The transgastric route was chosen for puncture because of a fresh ulcer in the duodenum. This route also offered a safer direction to puncture a nonobstructed CBD, which is nearer to the portal vein. A guidewire-negotiated antegrade into the duodenum revealed an inconspicuous ampullary opening at the edge of the ulcer (Fig. 5). A 5F nasopancreatic and a 6F nasobiliary drain were placed to divert the juices away from the ulcer to prevent delayed adverse events. The patient was administered 1 g flomoxef by intravenous route twice a day for 7 days.

## Outcome

**Figure 6:**
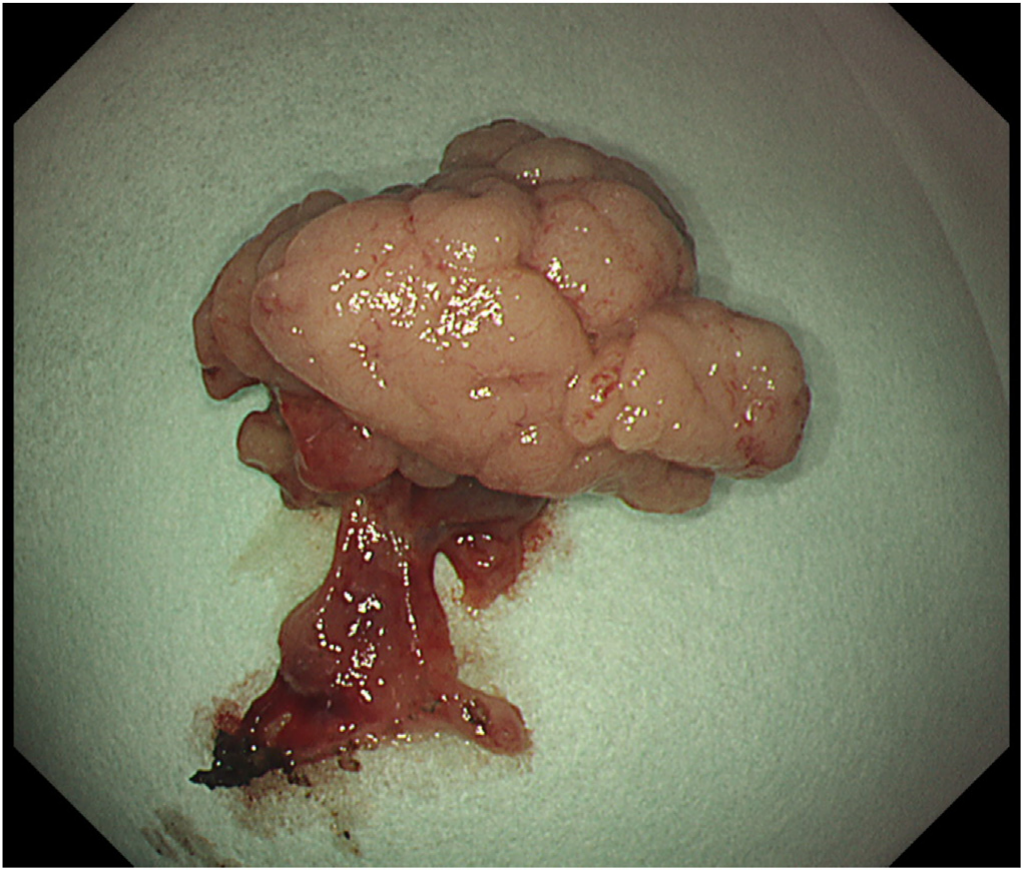
En bloc specimen (28 × 20 mm).

**Figure 7:**
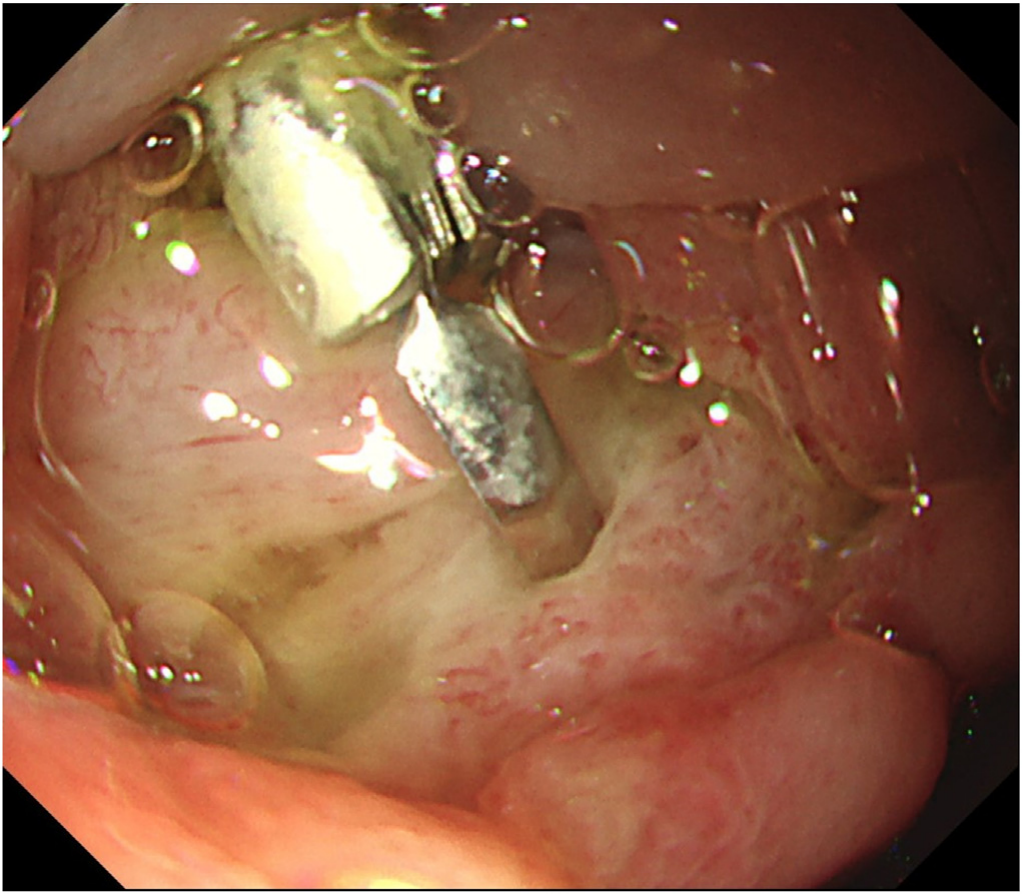
Postprocedure day 6: healing ulcer with healthy granulation tissue.

**Figure 8:**
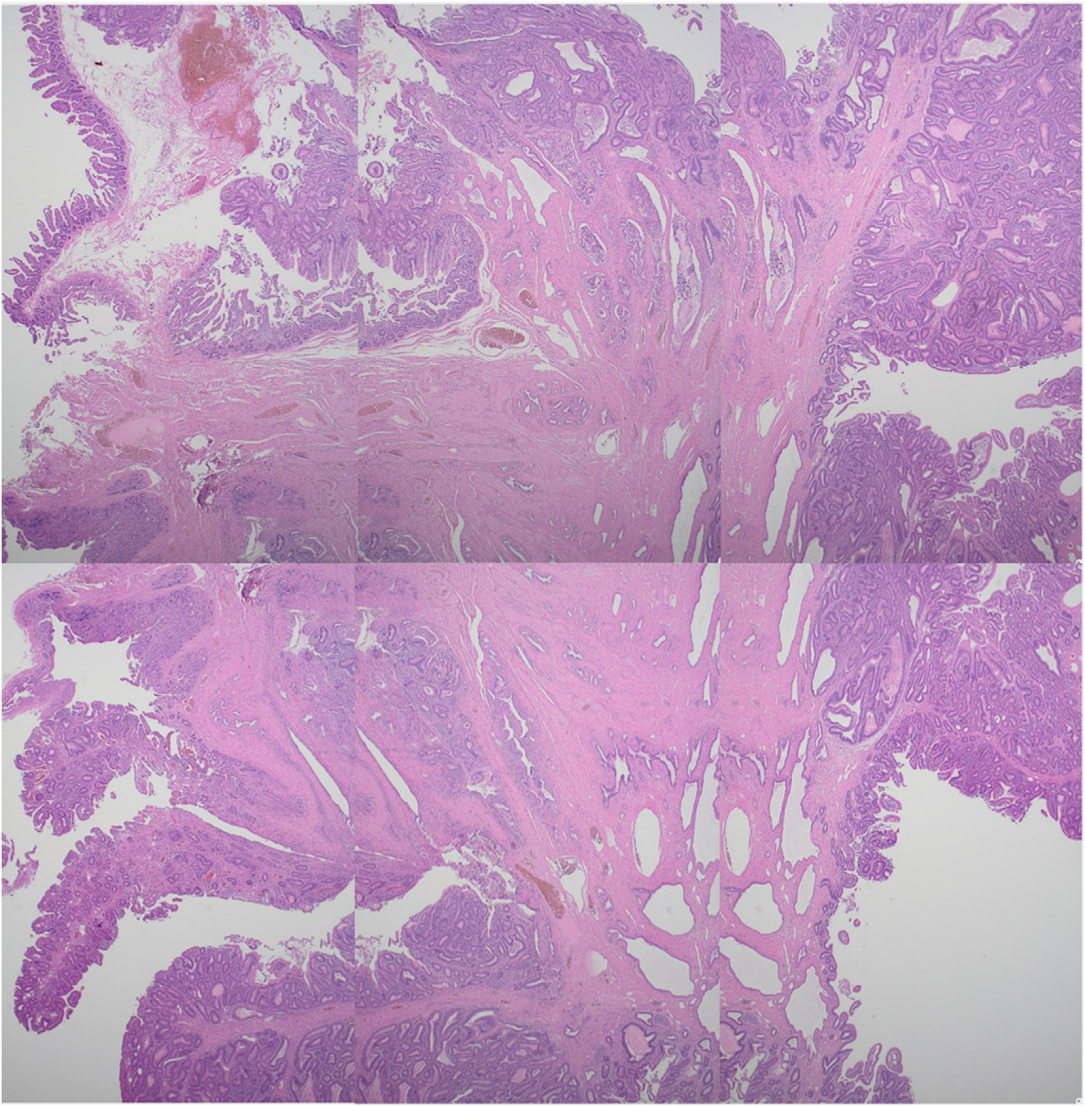
Histopathology: tubular adenoma with high-grade dysplasia. All margins negative (H&E, orig. mag. ×2).

Total procedure time (ESD + EUS-RV) was 50 minutes. The specimen measured 28 × 20 mm (Fig. 6). There were no immediate or early adverse events such as pain, bleeding, or pancreatitis. Endoscopy on day 6 revealed a healing ulcer with healthy granulation tissue (Fig. 7). Endonasal tubes were removed; the patient was started on a regular diet and discharged on day 6. Pathology was tubular adenoma with high-grade dysplasia with horizontal and vertical margins negative (Fig. 8).

## Conclusion

Endoscopic retrograde stenting is reported to be relatively easy after ampullary resection because of clearer identification of the opening.^5^ This case highlights an unfamiliar challenge of an obscured post-ESD ampullary opening, probably because of the opening being undermined below the edge of the ulcer. This shows a salvage use of EUS-RV for a complex case. Hence, expert technical skill and equipment in therapeutic EUS along with ESD are imperative while performing ampullary resections.

## Disclosure

The authors disclosed no financial relationships.

## Supplementary data

Video 1 EUS-Rendezvous post–ampullary ESD.

## References

[1] Kato M., Takeuchi Y., Hoteya S. (2022). Outcomes of endoscopic resection for superficial duodenal tumors: 10 years’ experience in 18 Japanese high volume centers. Endoscopy.

[2] Wang Y., Qi M., Hao Y., Hong J. (2019). The efficacy of prophylactic pancreatic stents against complications of post-endoscopic papillectomy or endoscopic ampullectomy: a systematic review and meta-analysis. Therap Adv Gastroenterol.

[3] Nakayama A., Kato M., Takatori Y. (2020). How I do it: endoscopic diagnosis for superficial non-ampullary duodenal epithelial tumors. Dig Endosc.

[4] Dhir V., Bhandari S., Bapat M., Maydeo A. (2012). Comparison of EUSguided rendezvous and precut papillotomy techniques for biliary access (with videos). Gastrointest Endosc.

[5] Spadaccini M., Fugazza A., Frazzoni L. (2020). Endoscopic papillectomy for neoplastic ampullary lesions: a systematic review with pooled analysis. United European Gastroenterol J.

